# Preoperative prediction of lymph node metastasis in endometrial cancer patients via an intratumoral and peritumoral multiparameter MRI radiomics nomogram

**DOI:** 10.3389/fonc.2024.1472892

**Published:** 2024-09-19

**Authors:** Bin Yan, Tingting Zhao, Ying Deng, Yili Zhang

**Affiliations:** ^1^ Department of Radiology, Shaanxi Provincial Tumor Hospital, Xi’an, China; ^2^ Department of Medical Imaging, First Affiliated Hospital of Xi’an Jiaotong University, Xi’an, Shaanxi, China; ^3^ Department of Medical Oncology, Shaanxi Provincial Tumor Hospital, Xi’an, China

**Keywords:** endometrial cancer, lymphatic metastasis, lymph node, magnetic resonance imaging, radiomics

## Abstract

**Introduction:**

While lymph node metastasis (LNM) plays a critical role in determining treatment options for endometrial cancer (EC) patients, the existing preoperative methods for evaluating the lymph node state are not always satisfactory. This study aimed to develop and validate a nomogram based on intra- and peritumoral radiomics features and multiparameter magnetic resonance imaging (MRI) features to preoperatively predict LNM in EC patients.

**Methods:**

Three hundred and seventy-four women with histologically confirmed EC were divided into training (n = 220), test (n = 94), and independent validation (n = 60) cohorts. Radiomic features were extracted from intra- and peritumoral regions via axial T2-weighted imaging (T2WI) and apparent diffusion coefficient (ADC) mapping. A radiomics model (annotated as the Radscore) was established using the selected features from different regions. The clinical parameters were statistically analyzed. A nomogram was developed by combining the Radscore and the most predictive clinical parameters. Decision curve analysis (DCA) and the net reclassification index (NRI) were used to assess the clinical benefit of using the nomogram.

**Results:**

Nine radiomics features were ultimately selected from the intra- and peritumoral regions via ADC mapping and T2WI. The nomogram combining the Radscore, serum CA125 level, and tumor area ratio achieved the highest AUCs in the training, test and independent validation sets (nomogram vs. Radscore vs. clinical model: 0.878 vs. 0.850 vs. 0.674 (training), 0.877 vs. 0.838 vs. 0.668 (test), and 0.864 vs. 0.836 vs. 0.618 (independent validation)). The DCA and NRI results revealed the nomogram had greater diagnostic performance and net clinical benefits than the Radscore alone.

**Conclusion:**

The combined intra- and peritumoral region multiparameter MRI radiomics nomogram showed good diagnostic performance and could be used to preoperatively predict LNM in patients with EC.

## 1 Introduction

Endometrial cancer (EC) is the most common type of gynecological malignancy in developed countries (1). Detecting lymph node metastasis (LNM) before surgery can influence the staging of EC patients, helping to guide the surgical strategy and plan adjuvant treatment. Systematic lymphadenectomy is routinely recommended according to the International Federation of Gynecology and Obstetrics (FIGO), yet its use remains controversial, particularly for low-risk disease (2), as there has been no demonstrated improvement in disease-free survival or overall survival for early-stage EC patients, regardless of the use of lymphadenectomy (3). Moreover, performing lymphadenectomy without discrimination could result in unnecessary treatment and an increase in postoperative complications such as infections, vascular/nerve damage, chronic lymphedema, and lymphocysts (4). Sentinel lymph node (SLN) mapping has been recommended for the intraoperative evaluation of LNM (5). A prior study concluded that sentinel node mapping is comparable to lymphadenectomy in identifying patients with nodal disease (6). More importantly, there is greater accuracy in detecting low-volume metastases (micrometastases and isolated tumor cells) through SLN mapping (6–10). Nevertheless, SLN mapping requires skilled surgeons (11). To carry out this process effectively, one must have access to new technology, indocyanine green tracers, and follow the SLN algorithm (12, 13). Hence, it is crucial in clinical practice to develop a noninvasive and easy method that can reliably predict the LN status of EC patients prior to surgery. This information will aid in tailoring personalized treatment strategies.

Magnetic resonance imaging (MRI) is crucial for evaluating deep myometrial invasion (DMI) in EC, but its ability to detect LNM is inadequate, with a sensitivity of 36.0%–89.5% (14). Previous research has indicated that various tumor morphological factors, such as the tumor volume (TV), tumor size (maximum diameter of the tumor, TS), tumor area ratio (TAR), and maximum anteroposterior tumor diameter on sagittal T2-weighted images (T2WIs) (APsag), are correlated with EC risk stratification (15–19). The serum cancer antigen 125 (CA125) level is a useful biomarker for predicting LNM in EC patients before surgery (20). Recently, radiomics studies utilizing intratumoral features have demonstrated positive outcomes in predicting LNM before surgery (21–24). Multiple studies evaluating various types of tumors have demonstrated the significance of radiomics features from the peritumoral margin in accurately predicting LNM before surgery (25–27). Nevertheless, there is a lack of research on enhancing the diagnostic accuracy of the preoperative prediction of LNM in EC through the integration of various tumor regions. Therefore, in this study, we aimed to develop and validate an MR-based radiomic nomogram combining different imaging sequences (i.e., apparent diffusion coefficient (ADC) mapping and T2-weighted imaging (T2WI)), different tumor regions (combined intra- and peritumoral regions), and different parameters (CA125 level, tumor morphological features, and radiomic features) for predicting LNM in patients with EC.

## 2 Materials and methods

### 2.1 Patients

**Figure 1:**
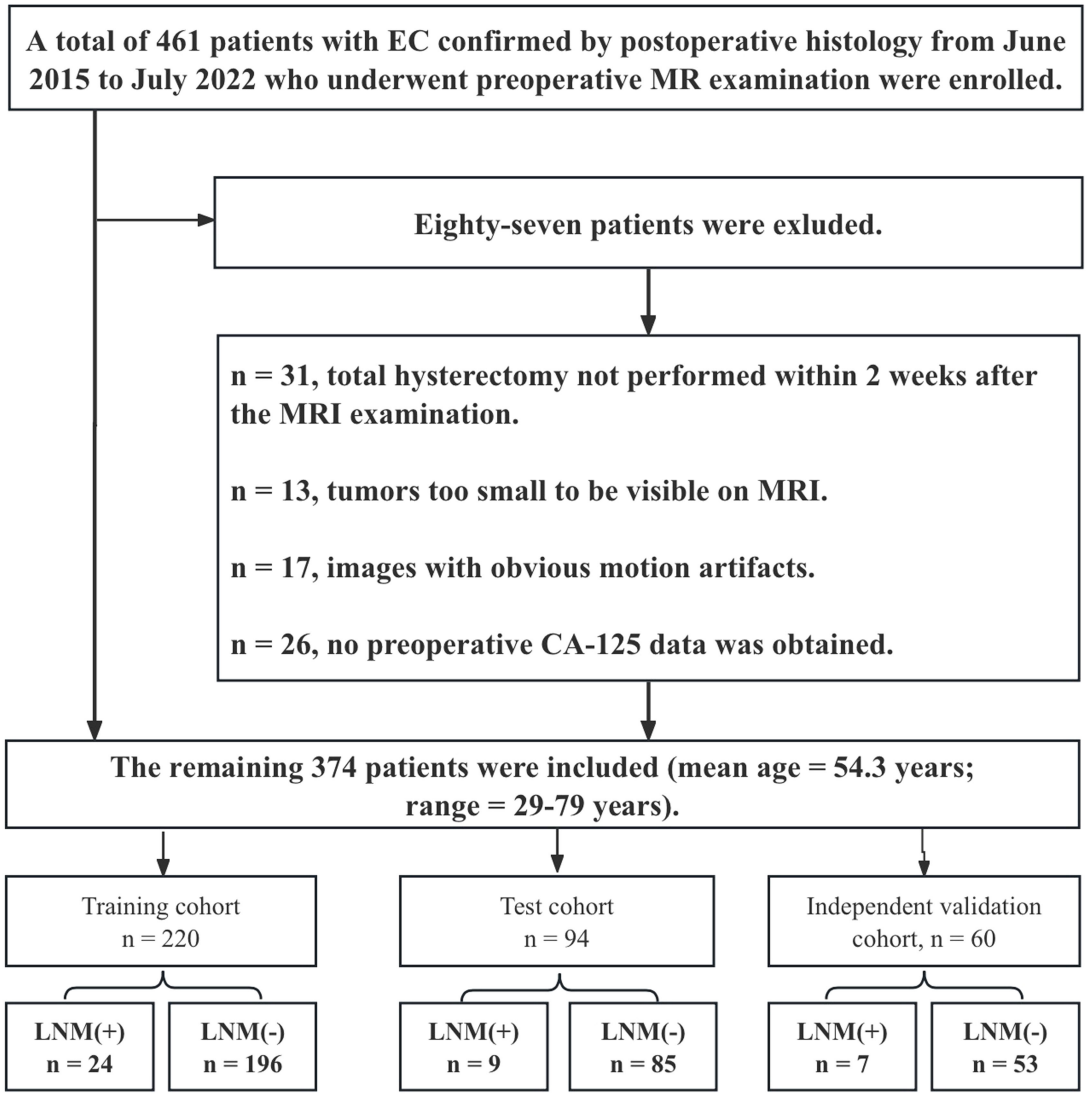
Flow chart demonstrating how the study population was chosen and the exclusion criteria applied.

This retrospective study was approved by the local ethics committee, and informed consent was not required. A total of 461 patients with EC confirmed by postoperative histology from June 2015 to July 2022 who underwent preoperative MR examination were enrolled. The inclusion criteria were as follows: (1) confirmation of EC through pathology; and (2) presence of clinical and histopathological characteristic data, including age, serum CA125 level, tumor grade, depth of myometrial invasion (MI), and cervical stromal invasion. The exclusion criteria were as follows: (1) total hysterectomy not performed within 2 weeks after the MRI examination; (2) preoperative treatment with chemoradiation; (3) tumors too small to be visible on MRI; (4) images with obvious motion artifacts; (5) contraindications for MRI examination; (6) incomplete clinical data; and (7) EC occurring simultaneously with other malignant tumors. Eighty-seven patients were excluded **Figure 1**), and the remaining 374 patients were included (mean age: 54.3 ± 8.1 years). Surgical staging of all patients included total hysterectomy with bilateral salpingo-oophorectomy, and lymph node (LN) assessment included pelvic lymphadenectomy and accompanying paraaortic lymphadenectomy. Compared with endometrioid adenocarcinoma (EEA), non-EEA (including carcinosarcomas, mucinous carcinoma, serous carcinoma, clear cell carcinoma, mixed carcinoma, and undifferentiated carcinoma) has a greater malignancy rate and is more prone to LNM (28). Therefore, we grouped non-EEA patients in similar proportions into training, test, and independent validation cohorts on the basis of the proportion of non-EEA patients in the overall cohort (10.7%, 40/374) (**Table 1**). Furthermore, patients with different field strengths were evaluated, with those who underwent 1.5-T MR accounting for 62.3% of the total cohort. Therefore, in the independent validation cohort, a similar proportion (61.6%) was also used for the 1.5-T dataset. The steps for data segmentation were as follows. First, 60 patients (37 patients who underwent 1.5-T MR and 23 who underwent 3.0-T MR) were randomly selected as the independent validation group. The remaining 314 patients were randomly divided into a training cohort (n = 220) and a test cohort (n = 94) at a ratio of 7:3. All patients were recruited from a single center. Notably, the independent validation cohort was not involved in the model training and testing phases.

**Table 1:** All patients’ clinical and tumor morphological parameters.

Characteristic	Training cohort (n = 220)		*P*	Test cohort (n = 94)		Independent-validation cohort (n = 60)	
	LNM(+) (n = 24)	LNM(-) (n = 196)		LNM(+) (n = 9)	LNM(-) (n = 85)	LNM(+) (n = 7)	LNM(-) (n = 53)
Age, years	54.0 ± 7.5	54.2 ± 8.0	0.861	55.1 ± 5.7	53.6 ± 7.6	59.1 ± 3.8	55.0 ± 10.0
CA125 (U/ml)	179.565 ± 443.404	39.810 ± 74.419	**0.001**	76.407 ± 65.676	35.577 ± 55.497	27.016 ± 17.808	43.940 ± 55.681
EEA	21	175		8	77	6	47
Non-EEA	3	21		1	8	1	6
Histological grade							
Grade 1 (G1)		14			3		7
Grade 2 (G2)	13	136		4	51	3	31
Grade 3 (G3)	11	46		5	31	4	15
Low-grade (G1+G2)	13	150		4	54	3	38
High-grade (G3)	11	46		5	31	4	15
MI							
Superficial	2	145		1	57	1	35
Deep	22	51		8	28	6	18
CSI							
Yes	13	38		5	17	4	7
No	11	158		4	68	3	46
Tumor volume, cm^3^	57.144 ± 88.535	18.213 ± 61.593	**0.000**	83.937 ± 120.469	13.932 ± 15.837	14.181 ± 9.266	23.446 ± 32.758
Tumor size, cm	5.893 ± 3.151	4.174 ± 2.323	**0.008**	6.982 ± 4.121	4.210 ± 1.807	4.694 ± 1.584	4.471 ± 2.017
APsag, cm	3.633 ± 2.033	1.909 ± 1.262	**0.000**	3.639 ± 2.325	2.059 ± 0.841	2.306 ± 0.608	2.328 ± 1.465
TAR, %	54.211 ± 27.402	31.410 ± 18.999	**0.000**	49.478 ± 17.740	35.808 ± 18.229	52.550 ± 20.444	35.088 ± 18.491
FIGO							
I		150			67		44
I a		117			45		32
I b		33			22		12
II		29			16		5
III	22	14		9	2	7	4
III a		12			2		4
III b		1					
III c1	16			5		6	
III c2	6	1		4		1	
IV	2	3					
IV a	1	3					
IV b	1						

The bold values indicates differences that are statistically significant. LNM, lymph node metastasis; EEA, endometrioid adenocarcinoma; Non-EEA, nonendometrioid adenocarcinoma (including mixed carcinoma, carcinosarcoma, undifferentiated carcinoma, serous carcinoma, and clear cell carcinoma); G1, well differentiated; G2, moderately differentiated; G3, poorly differentiated; low grade, G1 and G2; high grade, G3; CA125 = cancer antigen 125; MI, myometrial invasion; CSI, cervical stromal invasion; APsag, maximum anteroposterior diameter on sagittal T2W imaging; TAR, tumor area ratio; FIGO = International Federation of Obstetrics and Gynecology.

### 2.2 MRI protocol

The MRI scans were carried out via a 1.5-T (EXCELART Vantage™ powered by Atlas, Canon Medical Systems Corp., Tochigi, Japan) or 3.0-T (Siemens Magnetom Skyra, Erlangen, Germany) scanner, along with an 8-channel phased-array abdominal coil. To minimize artifacts resulting from intestinal motility, 20 mg of raceanisodamine hydrochloride (Hangzhou People’s Livelihood Pharmaceutical Co., Hangzhou, Zhejiang, China) was intravenously injected prior to scanning. All MRI sequences were obtained following a standard protocol (refer to **Supplementary Table 1** for specifics). For diffusion-weighted imaging (DWI), b = 0 and 650 s/mm^2^ were used for the 1.5-T scanner, and b = 0 and 1000 s/mm^2^ were used for the 3.0-T scanner. The ADC maps were automatically reconstructed by the postprocessing workstation.

### 2.3 Image preprocessing, image segmentation, and radiomics feature selection

**Figure 2:**
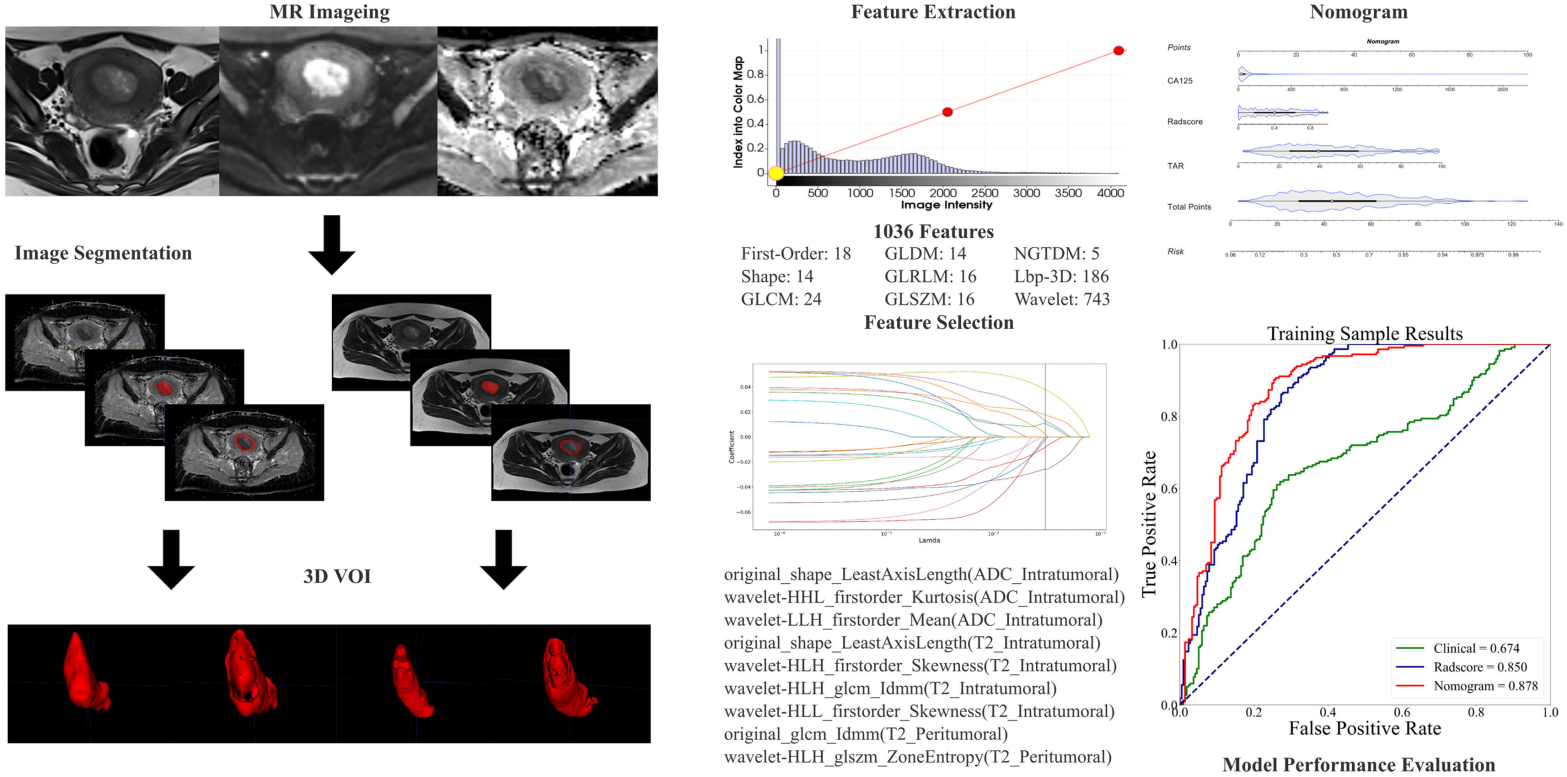
The radiomic workflow included manual segmentation of 3D VOIs from intra- and peritumoral regions, extraction of radiomic features, feature selection through LASSO regression, model development using nomograms, and evaluation of diagnostic performance via receiver operating characteristic (ROC) curve analysis.

Image preprocessing was performed via a standard workflow (see the **Supplementary Materials** for details). Image segmentation was performed by two experienced radiologists (T.Z. and B.Y.). One month after the initial segmentation, 100 patients were randomly selected for tumor segmentation by another radiologist (Y.D.) for interreader reliability evaluation. The details of the lesion segmentation are shown in the **Supplementary Materials**. 3D Slicer software (version 4.10.2; https://download.slicer.org/) was used for the manual whole-tumor segmentation. Three volumes of interest (VOIs) were chosen (**Figure 2**) as follows: (1) intratumoral region—ROIs were delineated along the edge of the lesion slice-by-slice, including areas of hemorrhage and necrosis, and the normal anatomical structure was avoided; the ROIs were fused into a three-dimensional (3D) VOI; (2) peritumoral margin—according to the previous literature (29), the tumor contour was automatically expanded by 3 mm to generate dilated VOIs; when the dilated VOIs were beyond the scope of the uterus, they were manually corrected for their boundaries to be set to the uterus edge; when other lesions in the myometrium (like fibroids, adenomyosis, etc.) were involved in the dilated VOI, manual correction was used; the peritumoral region = dilated VOI - tumor VOI; and (3) combined intra- and peritumoral regions.

The preprocessing of images and extraction of features were carried out via Artificial Intelligence Kit (AK, Version 3.3.0, GE Healthcare) software. Radiomics features, comprising first-order, shape-based, and texture features, were obtained from each of the VOIs. The methodology for extracting the radiomics parameters is shown in **Figure 2**.

### 2.4 Tumor morphological parameter measurements

**Figure 3:**
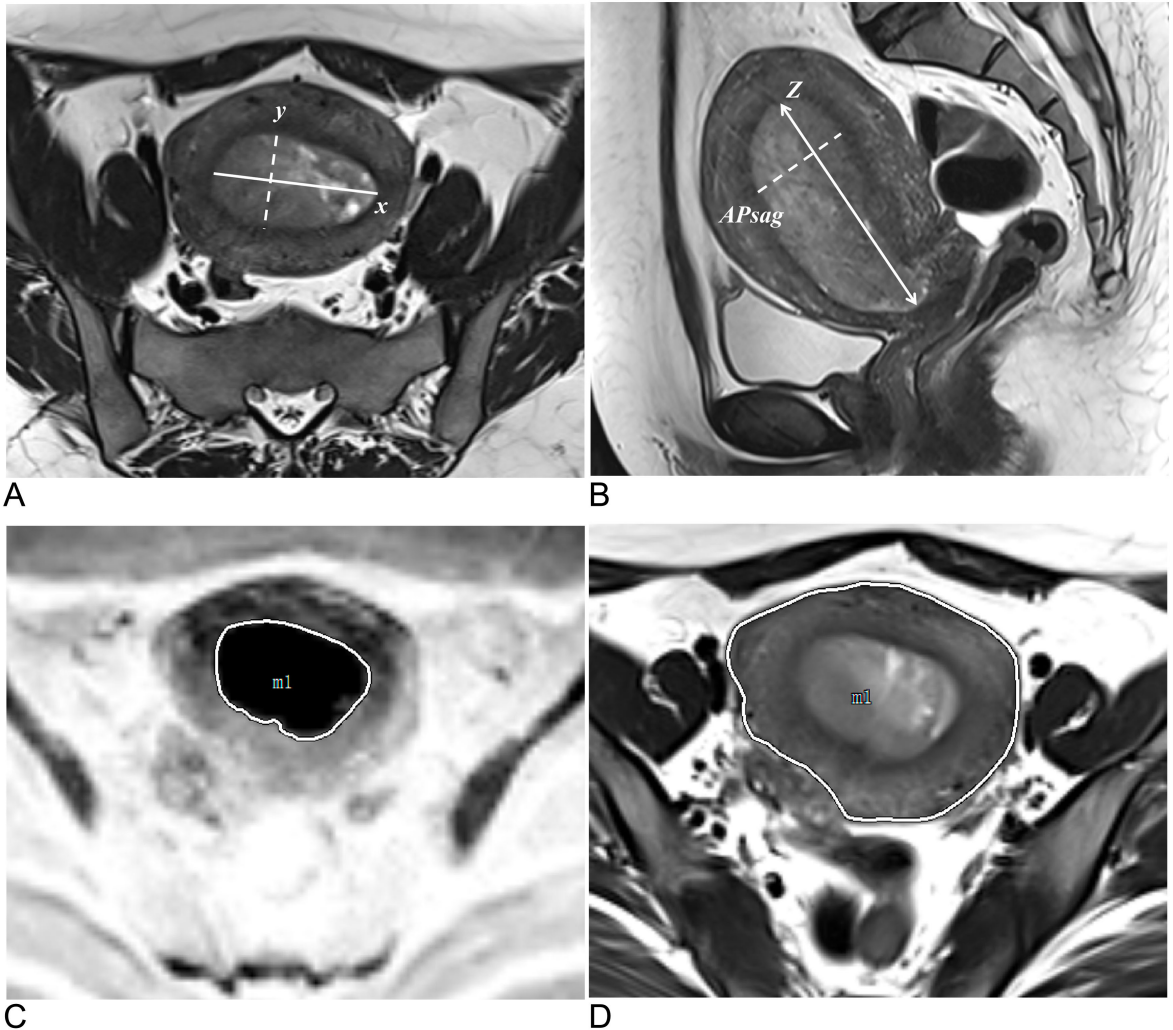
Methods for measuring tumor morphology parameters. **(A)** Measurements of the tumor’s maximum transverse diameter (solid line, x) and anteroposterior diameter (dotted line, y) were taken on oblique axial T2W images. **(B)** On sagittal T2W images, the tumor’s maximum craniocaudal diameter (solid line, z) and maximum anteroposterior diameter were measured (dotted line, APsag). **(C)** The tumor border on the DW image (reverse image) is shown by the white solid line. **(D)** The uterine border on the axial T2W image is depicted by the white solid line.

TS was defined as the maximum tumor diameter measured in three orthogonal planes: the transverse (*x*) and anteroposterior (*y*) diameters on oblique axial T2W images and the craniocaudal (*z*) diameter. APsag was measured on sagittal T2W images (**Figures 3A, B**). The whole-tumor volume on T2W images for the TV analysis was automatically calculated via 3D Slicer software. The TAR was calculated (the measurement method is detailed in the **Supplementary Material**) (**Figures 3C, D**) via the following equation from a previous study: TAR = (area of tumor/area of uterus) × 100% (18).

### 2.5 Statistical analysis

The statistical analysis was conducted via the R language (version 4.2.0, https://www.r-project.org) and Python language (version 3.9, https://www.python.org). A binary classification model was developed to predict the LNM status as “LNM-positive” or “LNM-negative”. Clinical data were subjected to both univariate and multivariate logistic regression (LR) analyses for filtration. Radiomic features were evaluated via t tests, Fisher’s exact tests, chi-square tests and, when applicable, the Mann−Whitney U test. *P <*0.05 was considered to indicate statistical significance. Least absolute shrinkage and selection operator (LASSO) regression was used to identify the most important radiomic features. LR was utilized for training the prediction models and was employed to create a nomogram incorporating clinical and tumor morphological parameters along with the radiomics score (Radscore). The independent validation cohort was used only to evaluate model performance. The sample size was calculated according to the previous literature (30); the number of events per variable was 10 or greater in the setting of the multivariate LR model. Before the initiation of data modeling, radiomic features were subjected to standardization via the standard scalar method. To determine the consistency among readers in evaluating MR morphological and radiomics features, intraclass correlation coefficients (ICCs) were calculated, with an ICC value exceeding 0.8 suggesting nearly perfect agreement. The R codes used for modeling and data analysis are provided in the **Supplementary Materials**.

Approximately 70% of EC patients are diagnosed with stage I disease (31). The risk of LNM in EC patients with low-grade and superficial MI is 3–5%, whereas the risk of LNM in high-grade patients is approximately 16–22% (32–34). Therefore, the proportion of LNM in EC is relatively low. In this study, approximately 10.7% (40/374) of patients had LNM. To address the imbalance between LNM-positive and LNM-negative patients in our training cohort, we employed the synthetic minority oversampling technique (SMOTE) to generate synthetic samples in the minority (positive) class. SMOTE works by selecting two or more similar instances (using a distance measure) in the minority class and generating new instances that lie between these instances in the feature space (35, 36). The SMOTE has been applied in previous studies on LNM in EC (24, 37).

The performance of the prediction model was evaluated via receiver operating characteristic (ROC) curve analysis. The DeLong test was employed to assess whether the difference in the ROC curves between the two models was statistically significant. The calibration curve was assessed via the Hosmer−Lemeshow (HL) test, with a *P* value greater than 0.05 indicating satisfactory predictive performance. Decision curve analysis (DCA) was used to compare the net benefits of the clinical models and radiomics nomogram models, with all ROC curve cutoff values determined by the maximum Youden index. The areas under the curve (AUCs), accuracy (ACC), sensitivity (SEN), and specificity (SPE) were then calculated.

## 3 Results

### 3.1 Patient clinical characteristics and MRI findings

**Table 1** summarizes the clinical and MRI morphological findings of the EC patients in the training, test, and independent validation cohorts. The 374 EC patients (1.5-T, n = 233; 3.0-T, n = 141) included 40 LNM-positive patients (40/374, 10.7%) and 334 LNM-negative patients. Forty non-EEA patients were included in this study (mixed carcinoma = 16, carcinosarcoma = 14, undifferentiated carcinoma = 4, serous carcinoma = 3, and clear cell carcinoma = 3) and were assigned to the training (24/220, 10.9%), test (9/94, 9.6%), and independent validation (7/60, 11.7%) cohorts in similar proportions.

### 3.2 Radiomics feature extraction, selection, and interreader reliability

**Figure 4:**
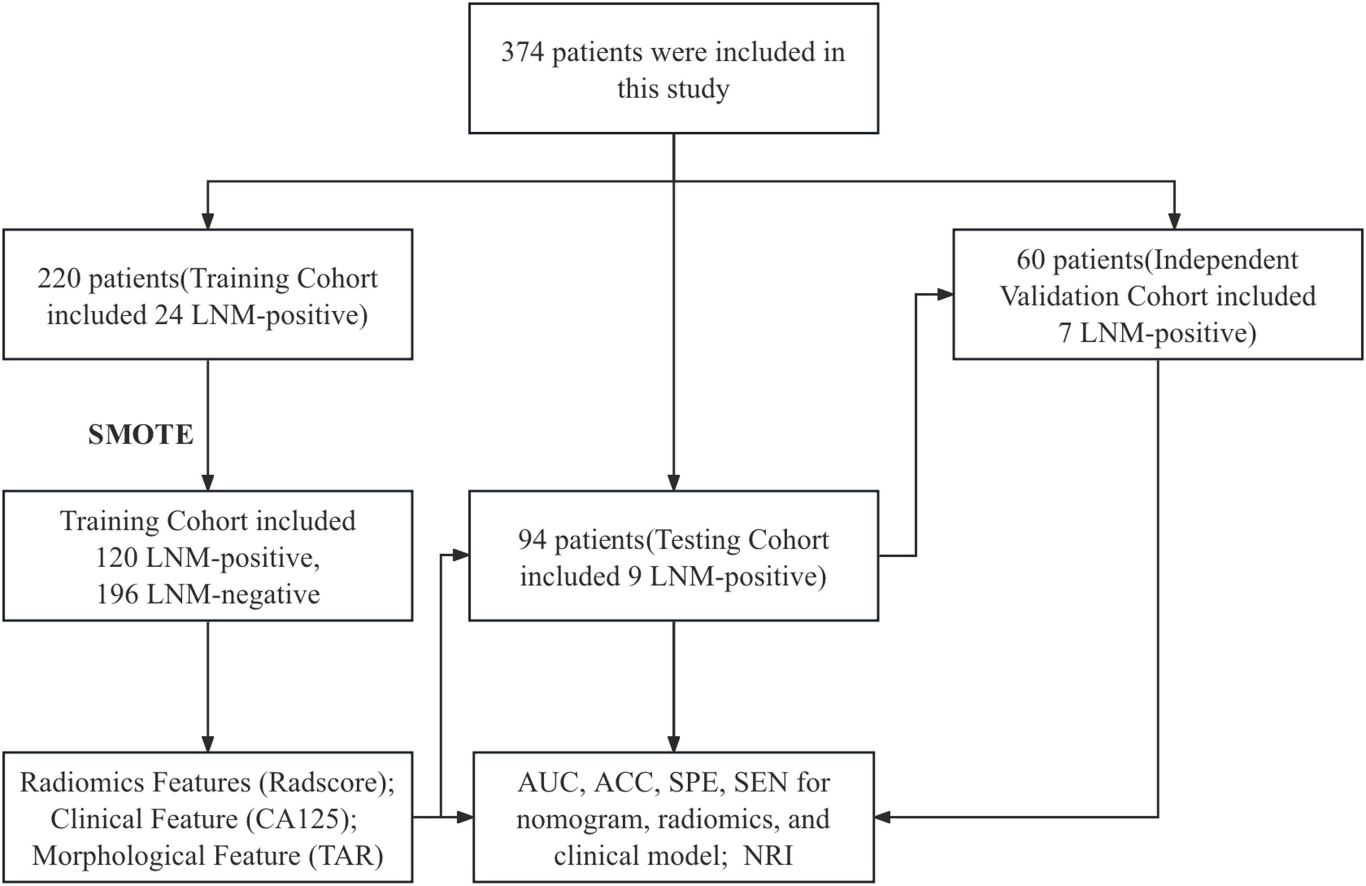
SMOTE workflow. AUC, area under the curve; ACC, accuracy; SPE, specificity; SEN, sensitivity; NRI, net reclassification improvement; LNM, lymph node metastasis; SMOTE. synthetic minority oversampling technique.

From each VOI, 1036 features were extracted, including ADC_Intratumoral, ADC_Peritumoral, T2WI_Intratumoral, and T2WI_Peritumoral. After feature selection, 9 features remained and were selected for evaluating LNM status in EC (forming the Radscore, **Table 2**) (refer to the **Supplementary Material** for a breakdown of the feature extraction process). There was no significant difference in the features between the training and test sets (**Table 3**). After the sample imbalance was adjusted with the SMOTE, the training cohort included 120 LNM-positive patients and 196 LNM-negative patients (**Figure 4**).

**Table 2:** Features forming the Radscore using the Logistic regression classifier.

Imaging	Region	Feature	*P* value
ADC mapping	Intratumoral	original_shape_LeastAxisLength	**0.002**
ADC mapping	Intratumoral	wavelet-HHL_firstorder_Kurtosis	**0.031**
ADC mapping	Intratumoral	wavelet-LLH_firstorder_Mean	**0.006**
T2-weighted imaging	Intratumoral	original_shape_LeastAxisLength	**<0.001**
T2-weighted imaging	Intratumoral	wavelet-HLH_firstorder_Skewness	**0.002**
T2-weighted imaging	Intratumoral	wavelet-HLH_glcm_Idmn	**0.029**
T2-weighted imaging	Intratumoral	wavelet-HLL_firstorder_Skewness	**<0.001**
T2-weighted imaging	Peritumoral	original_glcm_Idmn	**<0.001**
T2-weighted imaging	Peritumoral	wavelet-HLH_glszm_ZoneEntropy	**<0.001**

The bold values indicates differences that are statistically significant. ADC, apparent diffusion coefficient.

**Table 3:** Comparison of the Radiomics, clinical and morphological features in the training and test sets.

Features	*P value*
CA125	0.361
TAR	0.445
original_shape_LeastAxisLength (ADC_Intratumoral)	0.563
wavelet-HHL_firstorder_Kurtosis (ADC_Intratumoral)	0.276
wavelet-LLH_firstorder_Mean (ADC_Intratumoral)	0.631
original_shape_LeastAxisLength (T2_Intratumoral)	0.438
wavelet-HLH_firstorder_Skewness (T2_Intratumoral)	0.383
wavelet-HLH_glcm_Idmn (T2_Intratumoral)	0.426
wavelet-HLL_firstorder_Skewness (T2_Intratumoral)	0.503
original_glcm_Idmn (T2_Peritumoral)	0.389
wavelet-HLH_glszm_ZoneEntropy (T2_Peritumoral)	0.438

All the morphological parameters and radiomics features showed excellent interreader reliability, with ICC values ranging from 0.908 to 0.997. This high level of agreement ensures the repeatability of the model for future clinical use. The details are shown in **Supplementary Tables 4**-**6**.

### 3.3 Clinical model development and performance

After univariate analysis, the CA125 level, tumor grade, TV, TS, APsag, and TAR were significantly different between LNM-positive and LNM-negative patients (all *P* < 0.05, **Table 1**). Among these features, the tumor grade is a postoperative pathological result that can be obtained through preoperative endometrial biopsy or diagnostic curettage. Multivariate binary LR analysis revealed that the TAR and CA125 level were independent predictors of LNM in patients with EC (all *P* < 0.05, **Table 4**). The AUCs of the clinical model for predicting LNM in the training, test, and independent validation cohorts were 0.674 (95% confidence interval [CI]: 0.587–0.763; SEN: 68.4%, SPE: 58.7%), 0.668 (95% CI: 0.636–0.741; SEN: 75.0%, SPE: 64.2%) and 0.618 (95% CI: 0.567–0.654; SEN: 54.6%, SPE: 70.0%), respectively.

**Table 4:** Logistic regression analysis for predicting LNM positivity in patients with EC: the clinical model.

Parameters	Univariate			Multivariate		
	OR	95%CI	*p*	OR	95% CI	*p*
CA125	1.162	1.042-1.295	**0.007**	1.111	1.008-1.235	**0.050**
Tumor grade	10.476	2.696-40.698	**<0.001**	3.273	0.682-15.706	0.138
Tumor volume	1.371	1.152-1.630	**<0.001**	1.061	0.818-1.377	0.651
Tumor size	1.942	1.331-2.830	**<0.001**	0.833	0.385-1.801	0.643
APsag	3.428	2.160-5.439	**<0.001**	1.787	0.717-4.452	0.213
TAR	3.252	2.095-5.046	**<0.001**	2.056	1.104-3.828	**0.023**

The bold values indicates differences that are statistically significant. CI, confidence interval; OR, odds ratio; LNM, lymph node metastasis.

### 3.4 Radiomics model development and performance

The performance of ADC mapping and T2WI in distinguishing LNM-positive patients from LNM-negative patients is summarized in **Supplementary Table 7**. Interestingly, after applying LASSO regression with the peritumoral features from the ADC map, all feature coefficients decreased to zero. This implies that under the LASSO constraint, none of the features were identified as having substantial predictive power for the outcome. Therefore, ADC_Peritumoral features were excluded. Among the four radiomics models (ADC_Intratumoral, T2WI_Intratumoral, T2WI_Peritumoral, and T2WI_Intratumoral+Peritumoral), the combined intratumoral and peritumoral features from T2WI achieved the best prediction performance (AUC_training = 0.819; AUC_test = 0.771; and AUC_independent-validation = 0.772).

We combined the features of ADC mapping (intratumoral region) and T2WI (intra- and peritumoral regions) to construct the best prediction model (named hybrid-feature, **Table 5**), which showed the best classification performance (AUC_training = 0.850; AUC_test = 0.838; and AUC_independent-validation = 0.836). In this study, the Radscore consisted of 9 features from the hybrid-feature model.

**Table 5:** Features from different imaging sequences and VOIs combined to predict LNM.

Combined Model	Training cohort (n=220)				Test cohort (n=94)				Independent validation cohort (n = 60)			
	AUC 95% CI	ACC%	SPE%	SEN%	AUC 95% CI	ACC%	SPE%	SEN%	AUC 95% CI	ACC%	SPE%	SEN%
Model_1	0.844 (0.807-0.886)	74.2	73.7	74.7	0.821 (0.785-0.846)	74.7	75.4	66.7	0.811 (0.763-0.852)	78.7	78.6	80.0
Model_2	0.786 (0.725-0.822)	69.7	69.7	69.7	0.689 (0.605-0.733)	68.0	72.7	53.3	0.702 (0.649-0.732)	59.0	59.7	50.0
Model_3	0.850 (0.814-0.891)	78.5	73.2	83.8	0.838 (0.799-0.867)	72.0	71.0	83.3	0.836 (0.784-0.876)	72.1	71.4	80.0

Model_1, ADC_Intratumoral+T2WI_Intratumoral; Model_2, ADC_Intratumoral+T2WI_Peritumoral; Model_3, hybrid-feature (ADC_Intratumoral+T2WI_Intratumoral+T2WI_Peritumoral); AUC, area under the curve; CI, confidence interval; ACC, accuracy; SPE, specificity; SEN, sensitivity; VOI, volumes of interest; LNM, lymph node metastasis.

### 3.5 Diagnostic performance of the radiomic nomogram

**Figure 5:**
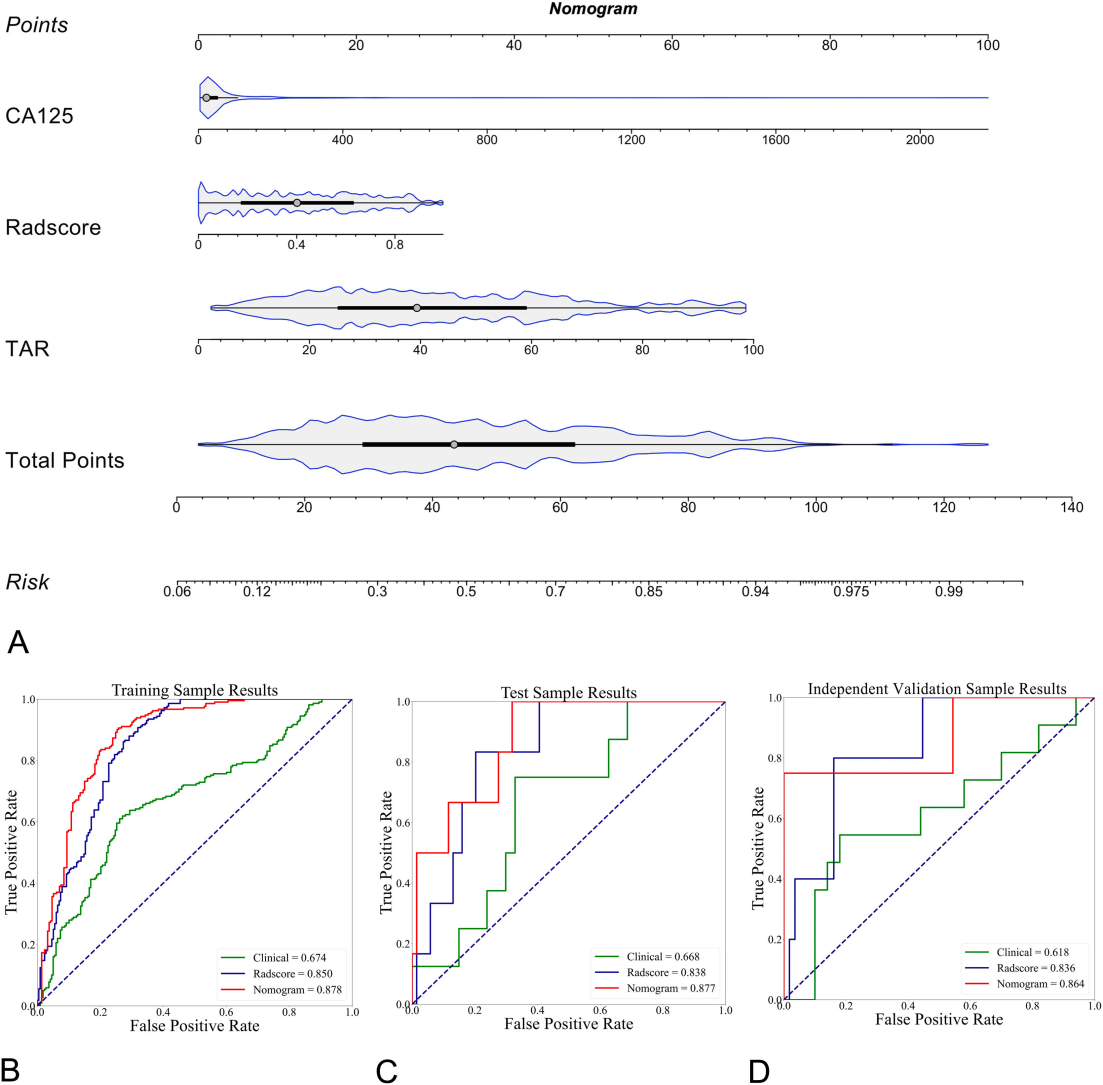
Explanation of the radiomics nomogram. **(A)** The nomogram of patients in the training cohort, which was developed by incorporating clinical (serum CA125 level) and morphological (TAR) features and the radiomics score (Radscore). According to this nomogram, the greater the risk is, the greater the likelihood that the patient will have LNM. **(B)** Comparison of ROC curves for different prediction models (n = 3) in differentiating LNM-positive and LNM-negative EC patients in the training cohort. The radiomics nomogram showed the highest AUC of 0.878 (95% CI, 0.823-0.907). In the test (**C**, AUC of 0.877, 95% CI: 0.831-0.914) and external validation (**D**, AUC of 0.864, 95% CI, 0.815-0.901) cohorts, the radiomics nomogram achieved the highest AUC.


The clinical model and the Radscore were combined, and a clinical–radiomics mixed model was constructed. The CA125 level, TAR, and Radscore were used to develop the nomogram in the training cohort (**Figure 5A**). The AUCs of the nomogram model for predicting LNM in the training (**Figure 5B**), test (**Figure 5C**) and independent validation (**Figure 5D**) cohorts were 0.878 (95% CI: 0.823–0.907; ACC: 81.0%, SEN: 84.0%, and SPE: 77.9%), 0.877 (95% CI: 0.831–0.914; ACC: 72.0%, SEN: 83.3%, and SPE: 71.0%) and 0.864 (95% CI: 0.815–0.901; ACC: 85.3%, SEN: 75.0%, and SPE: 86.0%), respectively. The formula was as follows:

$$\begin{array}{c} \text{Risk}=-1.68215933-0.025*\text{TAR}+0.001*\text{C}A125\\ \;\;\;\;+4.96239835*\text{Radscore} \end{array}$$

**Figure 6:**
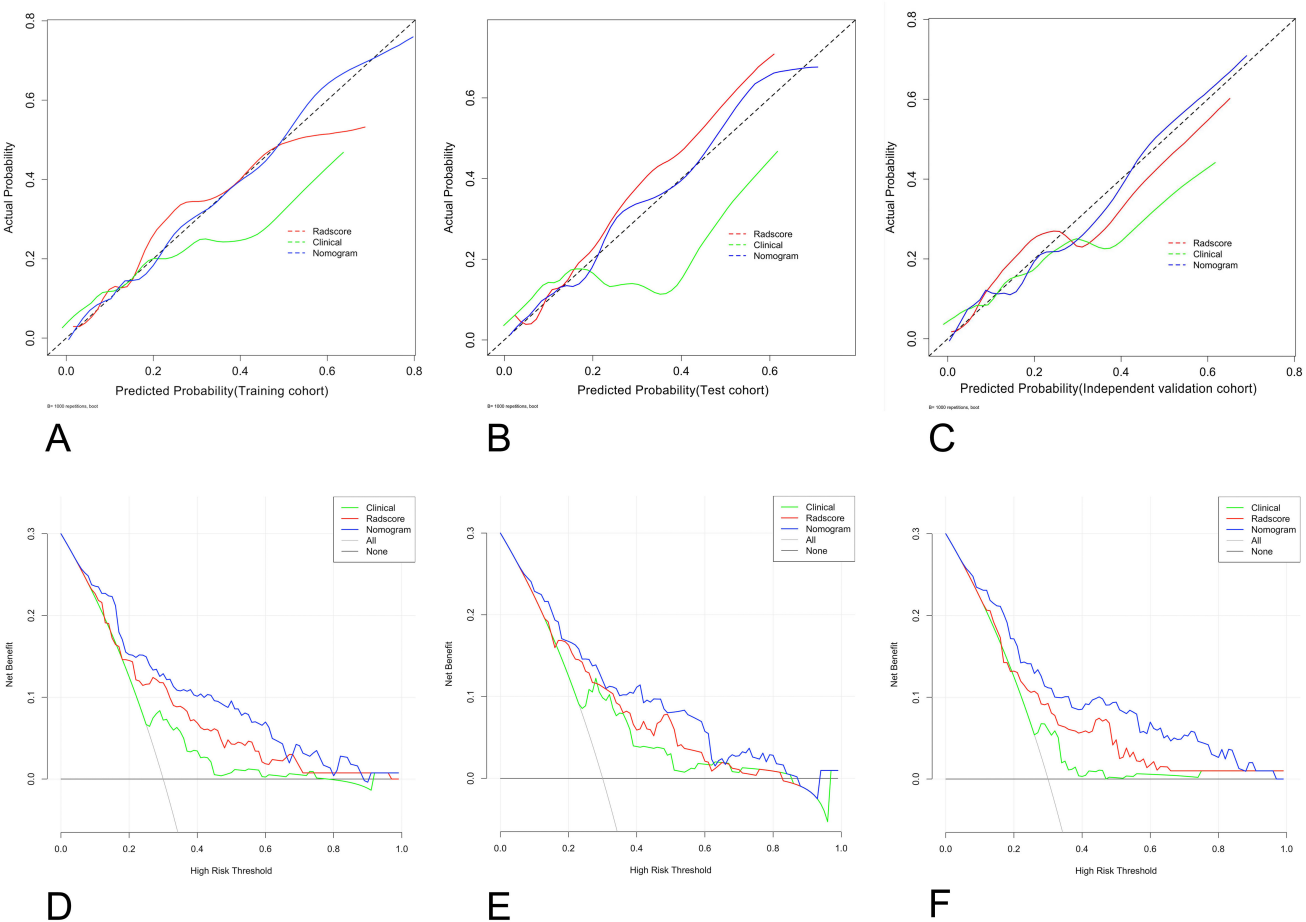
Graphs showing the calibration curves and decision curve analysis (DCA) results of the nomogram. **(A–C)** The calibration curves of the nomogram in the training **(A)**, test **(B)**, and external validation cohorts **(C)**; **(D–F)** for DCAs; the net benefit is on the vertical axis; the threshold probability is on the horizontal axis; the gray line represents the assumption that all patients are classified as having LNM; the black line represents the assumption that none of the patients are classified as having LNM; the green line represents the clinical model; the red line represents the radiomics score; the blue line represents the nomogram; and the DCA of the nomogram in the training **(D)**, test **(E)**, and external validation cohorts **(F)**. LNM, lymph node metastasis.

The calibration curves, with HL scores of 0.481, 0.346 and 0.226 for the training, test, and independent validation cohorts, respectively, revealed that the nomogram was reasonably accurate in predicting LNM in EC patients (**Figures 6A–C**). The DeLong test yielded a p value of 0.03 when the radiomics model was compared with the nomogram and a p value of 0.01 when the clinical model was compared with the nomogram. These results indicate that the differences in outcomes between the nomogram and both the radiomics and clinical models are statistically significant. DCA indicated that the radiomics nomogram produced greater net benefit than the clinical model for predicting LNM in EC patients in the training (**Figure 6D**), test (**Figure 6E**), and independent validation cohorts (**Figure 6F**). The reclassification measures confirmed that the nomogram performed better than the radiomics and clinical models did, with a net reclassification index (NRI) of 0.208 (95% CI, 0.112–0.287) when the nomogram and radiomics model were compared and an NRI of 0.386 (95% CI, 0.264–0.482) when the nomogram and clinical model were compared.

## 4 Discussion

In the present study, we developed and validated a brief radiomics nomogram for predicting LNM in patients with EC on the basis of the CA125 levels, TAR, and Radscore. This model showed good diagnostic performance (AUC_training = 0.878, AUC_test = 0.877, AUC_independent-validation = 0.864). Moreover, the features derived from distinct imaging sequences (i.e., T2WI and ADC mapping) and different VOIs (intratumoral, peritumoral, and combined regions) provided complementary information. Finally, a variety of field strength data were proportionally blended for the purpose of modeling and independent validation, thereby enhancing the real-world predictive capabilities of the models.

Radiomics uses automated and high-throughput feature extraction methods to transform images into feature data that can be mined. Currently, researchers have shown that intratumoral and peritumoral radiomics features in cancers such as lung, cervical, and breast cancers provide complementary information, which helps to improve LNM prediction model classification efficiency (38–40). In this study, the Radscore, which combines intra- and peritumoral features, achieved the best classification performance in predicting LNM (intratumoral region: AUC_ADC = 0.735, AUC_T2WI = 0.804; peritumoral region: AUC_T2WI = 0.770; and combined region: AUC = 0.850). This study revealed that the key features of the Radscore were wavelet transforms (WTs), which included histogram (skewness, kurtosis, and mean), texture (gray-level co-occurrence matrix (GLCM) and gray-level size zone matrix (GLSZM)) features. By utilizing the WTs, images are separated into high-frequency and low-frequency images for both intratumoral and peritumoral regions (41). The features calculated from the GLSZM offer insight into the heterogeneity of tumors, showing differences in the intensity and size of the homogeneous areas within regions (42). The GLCM is created by examining the connection between pairs of pixels and recording the occurrence of different gray-level combinations in an image or a region of interest. Compared with 2D ROIs, 3D VOIs have been shown to increase the specificity of GLCM features in identifying tumor components (43). Moreover, kurtosis and skewness are associated with high-risk histopathological features, such as lymphovascular space invasion (LVSI), DMI, and high-grade EC tumors in a previous study (44). Furthermore, the feature original_shape_LeastAxisLength was extracted from two image sequences (ADC mapping and T2WI) simultaneously, which has been repeatedly mentioned in a previous study of LNM classification in EC (37). Therefore, it is necessary to extract intra- and peritumoral radiomic features from ADC maps and T2W images to predict LNM in patients with EC.

In this study, the clinical model consisting of the CA125 level and TAR achieved moderate performance (AUC_training = 0.674, AUC_test = 0.668, and AUC_independent-validation = 0.618) in predicting LNM in patients with EC. To facilitate clinical application, we selected only tumor morphological parameters (i.e., TS, TV, TAR, and APsag) that can be obtained before surgery and are related to high-risk histopathological features (such as high-grade tumors, DMI, and LVSI) in EC (16–18, 45). After multivariate LR analysis, the CA125 level and TAR were found to be independent risk factors for predicting LNM in patients with EC. In previous studies, the CA125 level has been repeatedly mentioned as an independent risk factor for LNM in patients with EC (24, 46, 47). A previous study revealed that the TAR is closely related to DMI and high-grade tumors in patients with EC (18). To further improve the performance of the predictive model, we developed a nomogram that combines the clinical model and Radscore. The nomogram achieved good performance in predicting LNM, especially in the independent validation cohort (AUC = 0.864), proving that the model has high robustness. Moreover, due to the low rate of LNM positivity among our EC patients (40/374, 10.7%), the SMOTE was used to balance the dataset to improve the classification performance of the machine learning model. The model was further validated using a test set and an independent validation set, and the AUCs were 0.877 and 0.864, respectively, with no significant fluctuations. Additionally, DCAs and the reclassification measures of discrimination indicated that the radiomics nomograms achieved greater net benefit than did the clinical models in predicting LNM in patients with EC (NRI=0.386).

Previous studies have evaluated whether intratumoral radiomic features are useful for predicting LNM in patients with EC. Xu et al. (11) developed an MR-based radiomic nomogram (including the Radscore, LN size, and CA125 level) to predict LNM in normal-sized LNs, for which the AUCs were 0.892 and 0.883 in the training and test cohorts, respectively. Liu et al. (24) used an MR-based nomogram (including the Radscore, CA125 level, and MRI-reported MI) to predict LNM in patients with early-stage EC, and the AUCs in the training and test cohorts were 0.85 and 0.83, respectively. Our nomogram achieved similar predictive performance (AUC_training = 0.878, and AUC_test = 0.877) (11, 24). Compared with previous studies (11, 24), we established an independent validation cohort, and our nomogram achieved good predictive performance (AUC = 0.864), confirming that our model has good robustness. Second, we did not include information on MI depth, as, according to the previous literature, in some cases (such as when the junctional zone is unclear, when EC coexists with adenomyosis and/or uterine leiomyomas, or when the tumor is located in the area of the uterine cornua), it can be difficult to differentiate between superficial MI and DMI on the basis of MRI (48). Recently, Yan et al. (37) proposed radiologist-assisted MR radiomics for LNM in EC, which achieved good predictive performance (AUCs of 0.909 and 0.885 for validation sets 1 and 2, respectively). Interestingly, their study incorporated radiologists’ diagnoses of LNM, allowing some AI false-negative patients to be corrected in the final diagnosis. Although our AUCs did not exceed those of a previous study (37), grouping ECs with different histological subtypes in similar proportions to eliminate the impact of different histological subtypes on the results may be a potential strength.

This study has some potential advantages. (1) This study is the first to combine various tumor regions (combined intra- and peritumoral regions) and different parameters (clinical (CA125), tumor morphology (TAR), and radiomics features) to predict LNM in EC patients. The resulting model achieved good predictive performance and was validated across different field strengths. Tumor morphology characteristics are considered stable and do not change across scanners with different field strengths and manufacturers. Therefore, the radiomics nomogram, which combines the intra- and peritumoral features and incorporates both radiomics and morphological parameters, has better robustness and potential for clinical application. (2) Our prediction model will be beneficial for patients who are undergoing SLN mapping and who have negative pelvic LN on intraoperative frozen section analysis but may still require para-aortic lymphadenectomy. Previous studies have shown that the main obstacles in using SLN mapping are the detection rates and definition of para-aortic SLNs (49), as well as the risk of residual metastasis in non-SLNs (50). Consequently, our prediction model serves as a valuable complement to SLN mapping.

Our study has several limitations. First, no multicenter external validation was performed. Although we established separate validation cohorts to evaluate the robustness of the model, all the data were obtained from patients from a single center. A multicenter study of a larger dataset is needed to further validate the generalizability of our model. Second, this model included only the TAR and CA125 level; we did not investigate the LN size, which is an important criterion for MR to determine whether LNM is present. However, the sensitivity of the LN size in predicting LNM has been shown to be very poor (range, 36–89.5%) (14). If variables with very low sensitivity are included in an LR model, the implementation efficiency of the prediction model may be affected. Moreover, the influence of interreader variability on the LN size in MRI reports cannot be excluded. Therefore, we abandoned LN size measurements. Third, whole-tumor segmentation was manually performed instead of automatic/semiautomatic segmentation, which may be vulnerable to subjectivity and lead to inevitable bias despite our evaluation of interreader agreement. Fourth, including different pathological subtypes could have implications for predictive outcomes. EEA patients and non-EEA patients have different LNM risks. However, owing to the low incidence of LNM in EC, only 10.7% of patients in our study had LNM. Including only one subtype or stratifying by subtype could result in a small number of positive cases, which could affect the statistical performance of the radiomics model. Therefore, we decided not to use any specific pathological subtype as an inclusion criterion. As an alternative, we allocated EEA and non-EEA patients in similar proportions to the training, test, and validation cohorts to minimize the impact of different subtypes on our results. In the future, we plan to increase our sample size and develop predictive models specifically for each subtype to eliminate this confounding factor. Finally, this model is based solely on T2W and ADC images and does not consider contrast-enhanced T1W images. This could have potentially resulted in overlooking significant data. Despite these shortcomings, our study included an independent validation cohort, which decreased the risk of overfitting.

In conclusion, a combined intra- and peritumoral region multiparameter MRI radiomics nomogram was used to predict LNM in patients with EC. This model showed good diagnostic performance and may have potential clinical usefulness in the surgical treatment of EC patients. However, additional research is necessary to confirm its efficacy.

## Data Availability

The original contributions presented in the study are included in the article/**Supplementary Material**. Further inquiries can be directed to the corresponding authors.
