# Supplementary material for: Preoperative prediction of lymph node metastasis in endometrial cancer patients via an intratumoral and peritumoral multiparameter MRI radiomics nomogram
Source: Front Oncol. 2024 Sep 19;14:1472892. doi: 10.3389/fonc.2024.1472892 (PMC11446724; doi:10.3389/fonc.2024.1472892)
Supplement: Supplementary file 1 [file DataSheet1.docx]

Supplementary Material

# 2 Materials and methods

**2.2 MRI protocol**

Supplementary Table 1. Scanning sequence and parameters of the 1.5-T and 3.0-T MR scanners

| **Pulse Sequence** | **1.5-T MR** | **3.0-T MR** |
| --- | --- | --- |
| Axial T2WI | FRFSE, TR/TE = 4450/90 msec; matrix = 336 × 224; FOV = 280 × 400 mm; NEX = 2;  section thickness/intersection gap = 5/1.5 mm | TSE, TR/TE = 4290/93 msec; matrix = 384 × 345; FOV = 280×400 mm; NEX = 2;  section thickness/intersection gap = 5/1 mm |
| Axial T1WI | FSE, TR/TE = 605/15 msec; matrix = 336 × 224; FOV = 280 × 400 mm; NEX = 2;  section thickness/intersection gap = 5/1.5 mm | Dixon-VIBE, TR/TE = 5.55 /2.46 msec; matrix = 320 × 272; FOV = 280 × 400 mm; NEX = 1; section thickness/intersection gap = 4 /0.4 mm |
| Axial DWI | EPI, TR/TE/TI = 6900/80/150 ms;  b factors = 0 and 650 s/mm^2^; matrix = 128 × 128; FOV=280 × 400 mm; section thickness/intersection gap = 5/1.5 mm; speeder flag = 2; NEX = 4 | RESOLVE DWI, TR/TE = 5340/62 ms;  b factors = 0 and 1000 s/mm^2^;  matrix = 160 × 160; FOV = 280 × 400 mm;  section thickness/intersection gap = 5/1 mm; NEX = 3; readout segments = 5;  readout partial Fourier = 5/8. |
| Obligue T2WI | FRFSE, TR/TE = 2629/80 msec; matrix = 256 × 192; FOV = 250 × 200 mm; NEX = 2;  section thickness/intersection gap = 3.5/0.5 mm | TSE, TR/TE = 4750/108 msec; matrix = 320 × 224; FOV = 200 × 200 mm; NEX = 2;  section thickness/intersection gap = 3 /0.6 mm |
| Sagittal T2WI | FRFSE, TR/TE = 3324/90 msec; matrix = 320 × 224; FOV = 250 × 230 mm; NEX = 2;  section thickness/intersection gap = 4/1 mm | TSE, TR/TE = 6100 / 91 msec; matrix = 320 × 262; FOV = 250 × 230 mm; NEX = 2;  section thickness/intersection gap = 4 /0.4 mm |

DWI, diffusion-weighted imaging; EPI, echo-planar imaging; FSE, fast spin‒echo; FRFSE, fast-recovery fast spin‒echo; FOV, field of view; RESOLVE, readout segmentation of long variable echo-trains; TR, repetition time; TE, echo time; TI, inversion time; NEX, number of excitations; VIBE, volumetric interpolated breath-hold examination

**2.3 Image preprocessing, image segmentation, and radiomics feature selection**

**Image pre-processing** was performed using a standard workflow. Before the extraction of features, it is imperative to undertake a standardization process of the images. This involves several critical steps: firstly, resampling the spacing of all images to a uniform scale of [1.0, 1.0, 1.0]; secondly, normalizing the grayscale intensity across all images utilizing Z-score normalization techniques; and thirdly, employing Gaussian filtering to reduce noise within the images. These methodological procedures are essential to ensure the homogeneity and comparability of the dataset, which in turn significantly enhances the accuracy and reliability of subsequent feature extraction.

**Image segmentation** was independently performed by a radiologist with 5 years of experience in gynecological oncology MRI (reader_1, T.Z.) on axial T2W images and ADC images. Then, a radiologist with 10 years of experience in oncology MRI (reader_2, B.Y.) confirmed all tumor segmentations. When the two readers agreed, the segmentation result was retained. When two readers had different opinions, the final segmentation result was determined through discussion. All confirmed regions of interest (ROIs) were ultimately retained (reader_confirmed). One month after the initial segmentation, 100 patients were randomly selected for tumor segmentation by another radiologist with 8 years of experience in gynecological oncology MRI (reader_3, Y.D.) for interreader reliability evaluation.

**2.4 Tumor morphological parameter measurements**

The TAR was obtained through the following steps: (1) Measuring the tumor area. The radiologist selected the most representative slice, defined as the slice with the largest tumor area, and manually outlined the entire visible component with a single line on the DWI scan (Fig. 3c). (2) Determining the area of the uterus. The uterine area, defined on the same slice as the tumor area, was determined by delineating the serous surface of the uterus and manually delineating the entire visible component on the axial T2W image (Fig. 3d). (3) Defining the TAR. The TAR was obtained using the following equation: TAR = (area of tumor/area of uterus) × 100%.


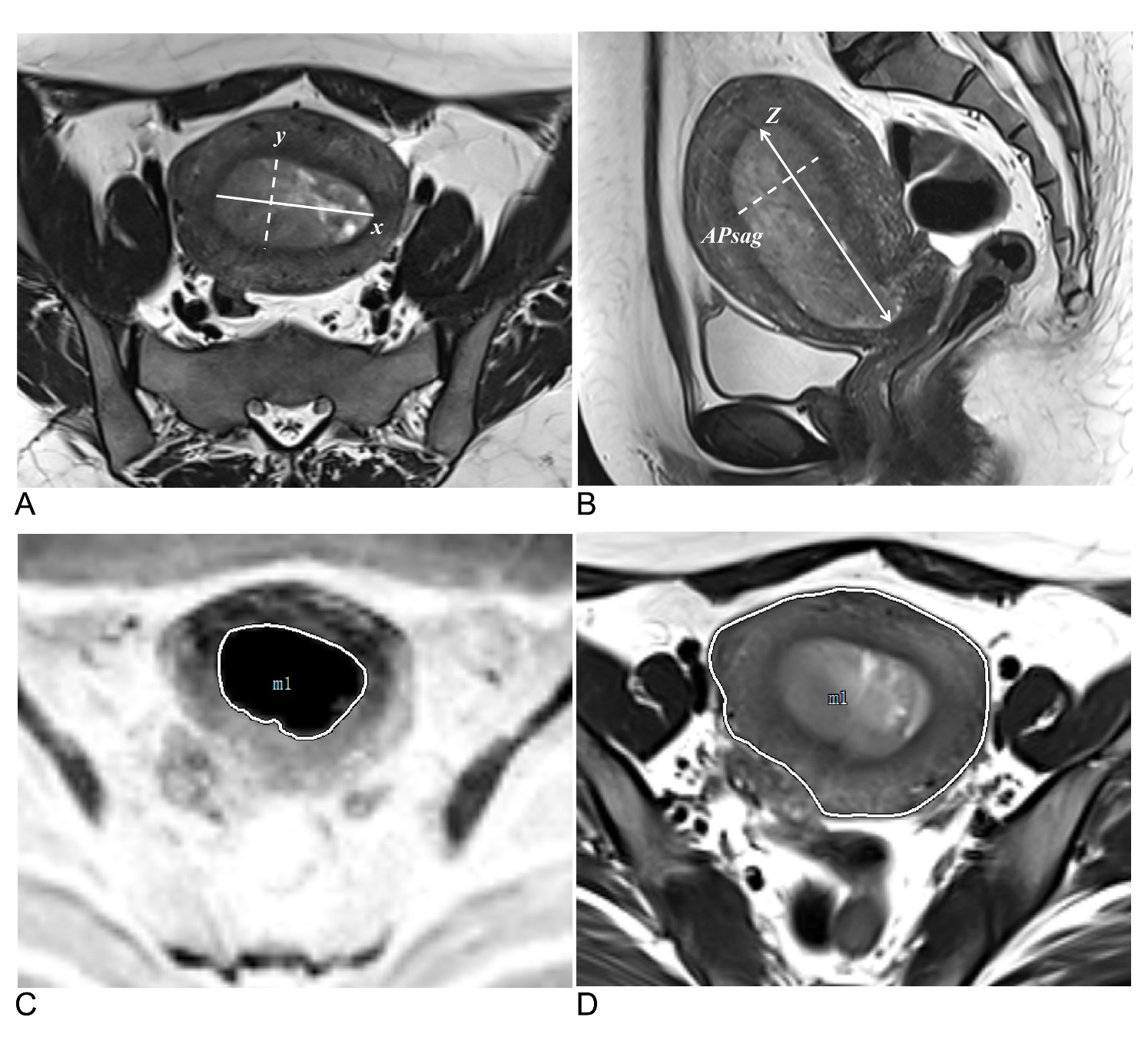


FIGURE 3. Methods for measuring tumor morphology parameters. (A) the maximum transverse diameter (solid line, *x*) and anteroposterior diameter (dotted line, *y*) of the tumor were measured on oblique axial T2W images; (B) the maximum craniocaudal diameter (solid line, *z*) and maximum anteroposterior diameter of the tumor were measured on sagittal T2W images (dotted line, APsag; (C) the white solid line indicates the tumor border on the DW image (reverse image); (D) the white solid line indicates the uterine border on the axial T2W image

**2.5 Statistical analysis**

The code used for modeling and data analysis is as follows.

For R software, the “glmnet” package was enlisted for the implementation of binary LASSO logistic regression and multivariate logistic regression with the objective of selecting pertinent radiomics and clinical features; the “rms” package was adopted for the execution of nomogram and calibration curve plotting; the “pROC” package was utilized for the calculation of the AUC; the “dca.R" package was invoked to carry out decision curve analysis; and the “PredictABEL” package was employed for the computation of the Net Reclassification Improvement (NRI).

**3 Results**

**3.2 Radiomics feature extraction, selection, and interreader reliability**

*Radiomics feature extraction and selection*

For each VOI (i.e., ADC_Intratumoral, ADC_Peritumoral, T2WII_ntratumoral, and T2WI_Peritumoral), 1036 radiomics features were extracted and then filtered by a preliminary feature selection process including the U test and correlation test. Features with *P* > 0.05 or correlation > 0.9 were deleted from the list of features. After feature removal, the number of features in the original data was reduced from 1036 to 78, 77, 85, and 67 in the ADC_Intratumoral, ADC_Peritumoral, T2WI_Intratumoral and T2WI_Peritumoral feature groups, respectively.

To select the most expressive features from the filtered data, the least absolute shrinkage and selection operator (LASSO) with 10-fold cross-validation was used. LASSO is the most appropriate method for searching for the most rewarding features in high-dimensional data. After LASSO regression, the number of features decreased from 78, 77, 85, and 67 to 9, 0, 7 and 6 in the ADC_Intratumoral, ADC_Peritumoral, T2WI_Intratumoral and T2WI_Peritumoral groups, respectively (detailed in Supplementary Table 2). Interestingly, after applying LASSO regression with the peritumoral features from the ADC map, all feature coefficients decreased to zero. This implies that under the LASSO regularization constraint, none of the features were identified as having substantial predictive power for the outcome.

To establish a model combining single-sequence intratumoral and peritumoral imaging features, we established the T2_features set by combining the 7 T2WI_Intratumoral and 6 T2WI_Peritumoral features. After LASSO regression, the number of T2 features was reduced from 13 to 8 (detailed in Supplementary Table 3).

To establish combined ADC-map and T2WI-feature models, the 9 ADC_Intratumoral and 7 T2WI_Intratumoral features were combined to establish a 16-feature set for creating Model_1; the 9 ADC_Intratumoral and 6 T2WI_Peritumoral features were combined to create a 15-feature set for establishing Model_2; and all 22 VOI features (ADC_Intratumoral + T2WI_Intratumoral + T2WI_Peritumoral) were combined to create the hybrid model (Model_3). After LASSO regression, the number of features of Model_1, Model_2, and Model_3 were reduced from 16, 15, and 22 to 9, 9, and 9 features, respectively (detailed in Supplementary Table 3).

The features of the ADC peritumoral region were excluded because all feature coefficients decreased to zero after LASSO regression. The remaining seven groups of features were used to build 7 logistic regression (LR) models, in which all features had nonzero coefficients.

In the hybrid-feature model, 9 features were included: 3 were from the ADC maps and 6 were from T2WI; of these, 7 were from intratumoral regions, and 2 were from peritumoral regions (detailed in Supplementary Table 3).

Supplementary Table 2. Radiomics feature selection procedures for each VOI and predicted model construction

| **Dataset** | **Selection Procedure** | | **Remain Features** | **Model** |
| --- | --- | --- | --- | --- |
| ADC_Intratumoral | Original | 1036 | original_shape_LeastAxisLength  log-sigma-3-0-mm-3D_firstorder_90Percentile  wavelet-HHL_firstorder_Kurtosis  wavelet-LLL_glszm_LargeAreaLowGrayLevelEmphasis  wavelet-LLH_firstorder_Mean  wavelet-HHH_gldm_LargeDependenceHighGrayLevelEmphasis  wavelet-HLL_firstorder_Kurtosis  wavelet-LHH_glszm_SmallAreaLowGrayLevelEmphasis  wavelet-LLH_glszm_SizeZoneNonUniformity | LR |
|  | U-test | 572 |  |  |
|  | Correlation | 78 |  |  |
|  | LASSO | 9 |  |  |
| ADC_Peritumoral | Original | 1036 | / | LR |
|  | U-test | 453 |  |  |
|  | Correlation | 77 |  |  |
|  | LASSO | 0 |  |  |
| T2WI_Intratumoral | Original | 1036 | original_shape_LeastAxisLength  wavelet-HLH_firstorder_Skewness  wavelet-HLH_glcm_Idmn  log-sigma-5-0-mm-3D_glcm_Imc1  wavelet-HHL_glszm_SmallAreaLowGrayLevelEmphasis  wavelet-HLL_firstorder_Skewness  log-sigma-3-0-mm-3D_glszm_GrayLevelNonUniformityNormalized | LR |
|  | U-test | 501 |  |  |
|  | Correlation | 85 |  |  |
|  | LASSO | 7 |  |  |
| T2WI_Peritumoral | Original | 1036 | wavelet-LLH_glrlm_LongRunEmphasis  original_gldm_LargeDependenceHighGrayLevelEmphasis  wavelet-LHH_glszm_GrayLevelNonUniformity  original_glcm_Idmn  wavelet-LHH_glszm_SizeZoneNonUniformity  wavelet-HLH_glszm_ZoneEntropy | LR |
|  | U-test | 325 |  |  |
|  | Correlation | 67 |  |  |
|  | LASSO | 6 |  |  |

LR, logistic regression

Supplementary Table 3. Combined features for the tumor feature experiment and predicted model construction

| **DataSet** | **Remain Features** | **Number** | **Model** |
| --- | --- | --- | --- |
| T2WI_Intratumoral +  T2WI_Peritumoral | Intratumoral_original_shape_LeastAxisLength  Intratumoral_wavelet-HLH_firstorder_Skewness  Intratumoral_wavelet-HLH_glcm_Idmn  Intratumoral_wavelet-HLL_firstorder_Skewness  Peritumoral_wavelet-HLH_glszm_ZoneEntropy  Peritumoral_wavelet-LHH_glszm_GrayLevelNonUniformity  Peritumoral_original_glcm_Idmn  Peritumoral_wavelet-LHH_glszm_SizeZoneNonUniformity | 8 | LR |
| Model_1:  ADC_Intratumoral +  T2WI_Intratumoral | ADC_original_shape_LeastAxisLength  ADC_wavelet-HHL_firstorder_Kurtosis  ADC_wavelet-LLH_firstorder_Mean  ADC_log-sigma-3-0-mm-3D_firstorder_90Percentile  ADC_wavelet-LHH_glszm_SmallAreaLowGrayLevelEmphasis  T2_original_shape_LeastAxisLength  T2_wavelet-HLH_firstorder_Skewness  T2_wavelet-HLH_glcm_Idmn  T2_wavelet-HLL_firstorder_Skewness | 9 | LR |
| Model_2:  ADC_Intratumoral +  T2WI_Peritumoral | ADC_original_shape_LeastAxisLength  ADC_wavelet-HHL_firstorder_Kurtosis  ADC_wavelet-LLH_firstorder_Mean  ADC_log-sigma-3-0-mm-3D_firstorder_90Percentile  ADC_wavelet-LHH_glszm_SmallAreaLowGrayLevelEmphasis  T2_wavelet-HLH_glszm_ZoneEntropy  T2_wavelet-LHH_glszm_GrayLevelNonUniformity  T2_original_glcm_Idmn  T2_wavelet-LHH_glszm_SizeZoneNonUniformity | 9 | LR |
| Model_3 (hybrid-feature):  ADC_Intratumoral +  T2WI_Intratumoral +  T2WI_Peritumoral | ADC_original_shape_LeastAxisLength  ADC_wavelet-HHL_firstorder_Kurtosis  ADC_wavelet-LLH_firstorder_Mean  T2_Intratumoral_original_shape_LeastAxisLength  T2_Intratumoral_wavelet-HLH_firstorder_Skewness  T2_Intratumoral_wavelet-HLH_glcm_Idmn  T2_Intratumoral_wavelet-HLL_firstorder_Skewness  T2_Peritumoral_original_glcm_Idmn  T2_Peritumoral_wavelet-HLH_glszm_ZoneEntropy | 9 | LR |

LR, logistic regression

*Interobserver reliability*

Excellent interreader reliability was obtained for all morphological parameters and radiomics features (ICC = 0.908‒0.997). The details are shown in Supplementary Tables 4-6.

Supplementary Table 4. Interreader variability of tumor measurements performed by three readers for 100 patients

| MRI morphological parameter | 1.5T-MR cohort (n=65) | 3.0T-MR cohort (n=35) |
| --- | --- | --- |
|  | ICC (95% CI) | ICC (95% CI) |
| Tumor volume (cm^3^) | 0.997 (0.995-0.998) | 0.973 (0.943-0.987) |
| APsag (cm) | 0.956 (0.925-0.973) | 0.943 (0.885-0.973) |
| Tumor size (cm) | 0.983 (0.971-0.989) | 0.934 (0.866-0.968) |
| Area of tumor (cm^2^) | 0.983 (0.970-0.989) | 0.969 (0.934-0.985) |
| Area of uterus (cm^2^) | 0.992 (0.988-0.996) | 0.961 (0.919-0.981) |

APsag, maximum anteroposterior tumor diameter on sagittal T2-weighted imaging

Supplementary Table 5. Interreader variability of radiomics features (intratumoral region) measured on ADC maps by three readers for 100 patients

| Radiomics feature (ADC mapping) | 1.5T-MR cohort (n=65) | 3.0T-MR cohort (n=35) |
| --- | --- | --- |
|  | ICC | ICC |
| original_shape_LeastAxisLength(ADC_Intratumoral) | 0.923(0.889-0.945) | 0.936(0.904-0.952) |
| log-sigma-3-0-mm-3D_firstorder_90Percentile(ADC_Intratumoral) | 0.997(0.982-0.999) | 0.907(0.887-0.921) |
| wavelet-HHL_firstorder_Kurtosis(ADC_Intratumoral) | 0.914(0.878-0.931) | 0.944(0.904-0.967) |
| wavelet-LLL_glszm_LargeAreaLowGrayLevelEmphasis(ADC_Intratumoral) | 0.963(0.942-0.988) | 0.952(0.936-0.971) |
| wavelet-LLH_firstorder_Mean(ADC_Intratumoral) | 0.932(0.913-0.951) | 0.961(0.944-0.973) |
| wavelet-HHH_gldm_LargeDependenceHighGrayLevelEmphasis(ADC_Intratumoral) | 0.908(0.886-0.926) | 0.952(0.930-0.967) |
| wavelet-HLL_firstorder_Kurtosis(ADC_Intratumoral) | 0.956(0.923-0.972) | 0.937(0.914-0.953) |
| wavelet-LHH_glszm_SmallAreaLowGrayLevelEmphasis(ADC_Intratumoral) | 0.988(0.945-0.992) | 0.918(0.896-0.944) |
| wavelet-LLH_glszm_SizeZoneNonUniformity(ADC_Intratumoral) | 0.946(0.922-0.960) | 0.933(0.911-0.945) |

Supplementary Table 6. Interreader variability of radiomics features (intratumoral region) measured on T2WI by three readers for 100 patients

| Radiomics feature (T2 mapping) | 1.5T-MR cohort (n=65) | 3.0T-MR cohort (n=35) |
| --- | --- | --- |
|  | ICC | ICC |
| original_shape_LeastAxisLength(T2_Intratumoral) | 0.942(0.922-0.960) | 0.965(0.943-0.973) |
| wavelet-HLH_firstorder_Skewness(T2_Intratumoral) | 0.935(0.923-0.952) | 0.921(0.903-0.935) |
| wavelet-HLH_glcm_Idmn(T2_Intratumoral) | 0.967(0.940-0.976) | 0.977(0.861-0.988) |
| log-sigma-5-0-mm-3D_glcm_Imc1(T2_Intratumoral) | 0.993(0.983-0.999) | 0.962(0.943-0.982) |
| wavelet-HHL_glszm_SmallAreaLowGrayLevelEmphasis(T2_Intratumoral) | 0.928(0.904-0.942) | 0.951(0.938-0.962) |
| wavelet-HLL_firstorder_Skewness(T2_Intratumoral) | 0.946(0.931-0.967) | 0.943(0.901-0.956) |
| log-sigma-3-0-mm-3D_glszm_GrayLevelNonUniformityNormalized(T2_Intratumoral) | 0.987(0.966-0.994) | 0.965(0.922-0.987) |

**3.4 Radiomics model development and performance**

Supplementary Table 7. Comparison of imaging features from two sequences and different VOIs

| **Imaging Sequence** | **Training cohort (n=220)** | | | | **Test cohort (n=94)** | | | | **Independent validation cohort (n = 60)** | | | |
| --- | --- | --- | --- | --- | --- | --- | --- | --- | --- | --- | --- | --- |
|  | AUC  95% CI | ACC% | SPE% | SEN% | AUC  95% CI | ACC% | SPE% | SEN% | AUC  95% CI | ACC% | SPE% | SEN% |
| ADC_Intratumoral | 0.735  (0.688-0.787) | 66.3 | 69.8 | 62.7 | 0.690  (0.621-0.744) | 62.7 | 65.2 | 44.4 | 0.707  (0.654-0.742) | 60.7 | 60.7 | 60.0 |
| T2WI_Intratumoral | 0.804  (0.765-0.834) | 71.7 | 67.7 | 75.6 | 0.733  (0.693-0.785) | 69.3 | 73.4 | 45.5 | 0.733  (0.689-0.775) | 65.6 | 66.0 | 62.5 |
| T2WI_Peritumoral | 0.770  (0.735-0.821) | 71.6 | 67.6 | 75.6 | 0.759  (0.714-0.803) | 69.3 | 67.2 | 87.5 | 0.685  (0.643-0.721) | 52.5 | 51.9 | 57.1 |
| T2WI_Intratumoral+  T2WI_Peritumoral | 0.819  (0.781-0.845) | 81.9 | 73.6 | 90.3 | 0.771  (0.729-0.804) | 66.7 | 66.7 | 66.7 | 0.772  (0.733-0.814) | 72.1 | 73.5 | 66.7 |

AUC, area under the curve; SPE, specificity; SEN, sensitivity; ACC, accuracy; CI, confidence interval; VOI, volume of interest.
